# Imidacloprid exposure in rats induces cardiac inflammatory response through activating TLR4/NF-κB/NLRP3 and JAK/STAT signaling pathways: focus on the berberine-loaded nanoliposomes

**DOI:** 10.3389/ftox.2025.1701021

**Published:** 2026-01-05

**Authors:** Layla Alkharashi, Amina A. Farag, Noha M. Gamil, Yasmen F. Mahran, Amira M. Badr, Heba Bayoumi, Mahmoud Mostafa, Awatif A. Binmughram, Aljawharah F. Alquhayz, Gadah M. BinObaid, Nervana M. Bayoumy, Eman E. Elwakeel, Reem T. Atawia

**Affiliations:** 1 Department of Pharmacology and Toxicology, College of Pharmacy, King Saud University, Riyadh, Saudi Arabia; 2 Department of Forensic Medicine and Clinical Toxicology, Faculty of Medicine, Benha University, Benha, Egypt; 3 Department of Pharmacology and Toxicology, College of Pharmaceutical Sciences and Drug Manufacturing, Misr University for Science and Technology (MUST), Giza, Egypt; 4 Department of Pharmacology and Toxicology, Faculty of Pharmacy, Ain Shams University, Cairo, Egypt; 5 Department of Histology and Cell Biology, Faculty of Medicine, Benha University, Benha, Egypt; 6 Histology department, School of Medicine, Badr Univesity in Cairo (BUC), Cairo, Egypt; 7 Department of Pharmaceutics, Faculty of Pharmacy, Minia University, Minia, Egypt; 8 Department of Pharmaceutics, Faculty of Pharmacy, Minia National University, Minia, Egypt; 9 Pharmacy College, King Saud University, Riyadh, Saudi Arabia; 10 Department of Physiology, College of Medicine, King Saud University, Riyadh, Saudi Arabia; 11 Anatomy and Embryology Department, Faculty of Medicine, Benha University, Benha, Egypt; 12 Department of Pharmaceutical Sciences, College of Pharmacy, Southwestern Oklahoma State University, Weatherford, OK, United States

**Keywords:** cardiotoxicity, imidacloprid, inflammasomes, JAK/STAT, berberine

## Abstract

The neonicotinoid insecticide, imidacloprid (IMI), is one of the widely used pesticides with well-documented serious health effects that are noticeable with long-term exposure. However, the long-term effects of IMI on cardiac tissues have not been fully elucidated. Herein, we investigated the mechanisms of IMI-induced cardiotoxicity. Additionally, we examined the potential protective effects of the natural alkaloid, berberine (BBR), against IMI-induced cardiotoxicity. Rats received IMI (45 mg/kg/day, orally) for 30 days, alone or in combination with BBR-loaded liposomes (BBR-Lip) at a dose of 10 mg/kg, intraperitoneally. Cardiac troponin I (cTnI), creatine kinase-MB (CK-MB), oxidative stress, inflammatory markers, and histopathological alterations were assessed. IMI caused significant cardiac damage as shown by increased levels of cTnI and CK-MB and histopathological insults examined by H and E and transmission electron microscopy. These changes were accompanied by the induction of oxidative stress and inflammatory markers. Additionally, IMI inhibited the expression of Nrf2, a powerful regulator of cellular antioxidant defense and activated inflammatory pathways by inducing expressions of TLR-4, NF-κB, NLRP3-inflammasome and gasdermin. Moreover, IMI induced cardiac expressions of TGF-β, p-JAK, and p-STAT, which worsens the oxidative stress and inflammatory status. Co-administration of BBR-Lip attenuated the biochemical, histological and molecular dysregulation induced by IMI in cardiac tissues. Collectively, this study provides mechanistic insights into the cardiotoxic effects of IMI as well as the potential protective effects of BBR-Lip.

## Graphical Abstract

**Figure:**
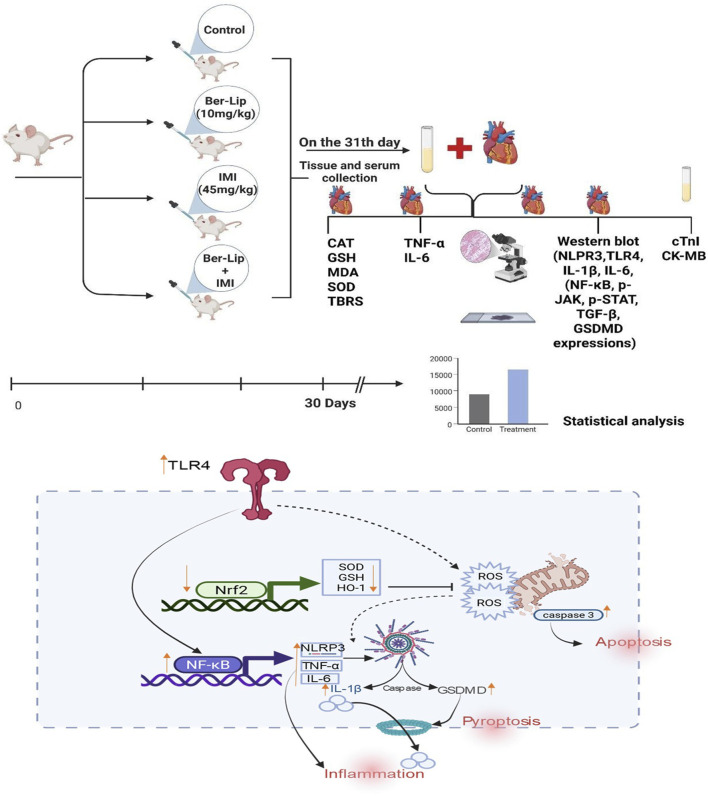

## 1 Introduction

Imidacloprid (IMI) is a neonicotinoid insecticide that is commonly used for both agricultural and veterinary purposes (Jeschke et al., 2011). Nevertheless, its presence in water, soil, and living organisms, resulting from long half-lives, high solubility in water, and low soil sorption, raise substantial concerns for environmental health (Goulson, 2013). Human exposure to IMI mainly occurs through food, drinking water, occupational settings, and spray drift (Katić et al., 2021). Long-term exposure to IMI has been shown to cause various health effects, including endocrine disruption, hepatotoxicity, nephrotoxicity, and neurotoxicity (Katić et al., 2021). In addition, incidents of acute poisoning and deaths have also been documented. Despite evidence of deleterious cardiovascular effects associated with IMI exposure in clinical settings (Shadnia and Moghaddam, 2008; Huang et al., 2006), the exact mechanism of IMI-induced cardiotoxicity in mammals is not clearly understood.

IMI toxicity was found to be linked to the induction of reactive oxygen species (ROS) and the disruption of antioxidants, which finally results in redox imbalance and induction of oxidative stress (Lesser, 2006). This oxidative burden triggers the release and subsequent nuclear translocation of the well-known antioxidant regulator nuclear factor erythroid 2-related Factor 2 (Nrf2). Nrf2 induces the expression of several antioxidant genes, such as the one coding for hemeoxygenase-1 (Abais et al., 2014). Excess ROS is associated with the induction of various inflammatory and apoptotic pathways. One of the crucial pathways activated by ROS is the Nod-like Receptor family pyrin domain containing 3 (NLRP3) inflammasome pathway (Abais et al., 2014), which is linked to poor prognosis of heart diseases and adverse cardiovascular effects (Toldo et al., 2022). Additionally, Janus kinase-signal transducer and activator of transcription (JAK-STAT) signaling pathway, which is crucial for several biological processes and cellular homeostasis (Guo et al., 2024), is involved in the intricate cross-talk between oxidative stress and inflammation (López-Hernández et al., 2025). Persistent inflammation can induce different forms of cell death, such as pyroptosis, mediated through NLRP3 inflammsome/caspase-1 activation of gasdermin (GASDMD) (López-Hernández et al., 2025), and apoptosis, which can be induced by different insults culminating in the activation of the cell death executive protein; caspase-3 (García-Muñoz et al., 2024).

Berberine (BBR) is a naturally occurring isoquinoline alkaloid from the plant *Coptis chinensis*, with a wide array of biological properties and therapeutic applications. These include anti-inflammatory, antitumor, antioxidant, and anti-apoptotic effects (García-Muñoz et al., 2024; An, 2022). BBR also showed promising cardioprotective and antioxidant activities (Wang et al., 2023). However, the effect of BBR on IMI-induced cardiotoxicity and its ability to modulate cardiac inflammasomes and JAK/STAT pathways, has not been fully elucidated.

In this study, BBR-loaded nanoliposomes (BBR-Lip) were used to improve its bioavailability and targeted delivery. Nanoliposomes may potentially limit off-target side effects by ensuring that a greater proportion of the BBR reaches the cardiac tissues where it is most needed, while simultaneously reducing systemic exposure (Vanti et al., 2021; Duong et al., 2021). Based on the information mentioned above, we hypothesize that imidacloprid causes cardiotoxicity via oxidative stress and inflammation, involving TLR4/NF-κB/NLRP3 inflammasome and JAK/STAT pathways, leading to cardiomyocyte dysfunction, apoptosis, and pyroptosis. We also hypothesize that BBR-loaded nanoliposomes (BBR-Lip) protect the heart by reducing oxidative damage through Nrf2 activation, suppressing inflammation, modulating TLR4/NF-κB/NLRP3 and JAK/STAT pathways, and maintaining cardiac integrity and function.

## 2 Materials and methods

### 2.1 Insecticide (imidacloprid; IMI), supplements, and chemicals

IMI (N-[1-[(6-chloropyridin-3-yl) methyl]-4,5-dihydroimidazol-2-yl nitramide), a technical-grade white powder with ≥96% purity (Top Chemicals, China), was generously provided by the Central Agricultural Pesticide Laboratories in Giza, Egypt. BBR was obtained from Sigma-Aldrich (St. Louis, MO, United States). Phospholipon 90G was gifted by Lipoid GmbH (Ludwigshafen, Germany). Cholesterol was purchased from Fluka Chemicals (NC, United States). Antibodies targeting toll-like receptor-4 (TLR4, Cat. # ab22048), nuclear factor-κB (NF-κBp65, Cat. # ab16502), transforming growth factor-β1 (TGF β1, Cat. # ab64715), NLRP3 (Cat. # ab263899), IL-1β (Cat. # ab254360), gasdermin (GSDMD, Cat. # ab219800), and glyceraldehyde-3-phosphate dehydrogenase (GAPDH, Cat.# ab8245) were sourced from Abcam Inc., Cambridge, MA, United States. All other chemicals were of high analytical grade.

### 2.2 Preparation of BBR chloride-loaded PEGylated nanoliposomes

BBR-Lip were prepared using the solvent injection method, as previously described (Khater et al., 2023; Mansour et al., 2025). Briefly, the lipid components (Phospholipon 90G and cholesterol) and BBR were accurately weighed and dissolved in anhydrous ethanol to form a uniform lipid phase. This lipid solution was then slowly injected into the aqueous phase containing PEG_4000_ under continuous magnetic stirring. The rapid diffusion of the organic solvent into the aqueous medium promoted the self-assembly of lipids into nano-sized liposomal vesicles encapsulating BBR. The organic solvent was subsequently removed by gentle evaporation, and the resulting liposomal dispersion was further homogenized and filtered to achieve size uniformity. The final BBR-Lip formulation was stored at 4 °C until further characterization and use. The berberine-loaded liposomal formulation used in this work is the same previously characterized batch reported by El Gazzar et al. (2025).

### 2.3 Animals

Thirty-two adult male Sprague–Dawley rats weighing between 150 and 180 g were obtained from the animal house colony of the Faculty of Science, Benha University, Egypt. During the experiments, animals were kept in well-ventilated cages in groups of three animals per cage. Before the experiments began, they were allowed to acclimate for a week. Rats were housed under standardized conditions with adequate ventilation, temperature (23 °C ± 2 °C), and a 12-h light-dark cycle. The rats were fed a standard diet of known composition and water *ad libitum.* Procedures involving animals and their care were approved by the Research Ethics Committee of the Faculty of Pharmacy, Minia University, Egypt (Approval code MPEC 2401105). Animals were treated in accordance with the guidelines and ethics of the Animal Protocol following ARRIVE guidelines.

### 2.4 Experimental design

The rats were randomly allocated into four groups, each comprising eight rats, as follows:

Group I received corn oil, PO, as the vehicle. Group II received corn oil PO, and was treated with BBR-Lip (10 mg/kg) intraperitoneal (i.p.) (El Gazzar et al., 2025). Group III received IMI 45 mg/kg (1/10 of LD50, i.e. 1/10 × 450 mg/kg). IMI was suspended in corn oil and given PO (El Gazzar et al., 2025; Abbassy, 2014). IMI was suspended in corn oil as a vehicle. Due to the limited lipophilicity of IMI (Log = 0.57), the compound forms a suspension rather than a proper solution in corn oil. To ensure uniform distribution and consistent dosing, the corn oil suspension was vortexed for 2 min immediately before oral gavage (PO) administration using a tuberculin syringe with a soft rubber catheter.

Rats in group IV were treated with IMI (45 mg/kg PO) and BBR-Lip (10 mg/kg i. p.). BBR-Lip and IMI were given daily for 30 days. The dose of IMI was selected based on earlier reports (El Gazzar et al., 2025; Abbassy, 2014) and on a pilot study in our lab using two different doses 22.5 and 45 mg/kg. The data of histology and cardiac enzymes showed no significant toxic effect of the low dose. So, the dose of 45 mg/kg was selected for the current research. (Supplementary Table S1).

All rats underwent inhalation euthanasia with isoflurane on the thirty-first day. Rats were exposed to 5% isoflurane in plexiglass chamber for 5 min, in which rats were sedated and lost paw reflex, at that point blood samples were obtained from the abdominal aorta, sera were isolated, aliquoted, and kept at −80 °C for biochemical analyses. This was followed by decapitation, and the hearts were then removed. A portion of the tissues was homogenized in phosphate-buffered saline, pH 7.4 and centrifuged at 1.07 × 10^5^ × g for 15 min at 4 °C. The supernatant was used to study the biochemical parameters in heart tissue samples. The ventricles of the heart were processed for histopathological examination, transmission electron microscopy (TEM), immunohistochemical staining, and Western blotting.

### 2.5 Histological study

The histological features were examined using hematoxylin and eosin (H and E) as described previously (Bancroft et al., 2019). In brief, after rats were sacrificed, paraffin blocks were prepared for the isolated left ventricular tissues to assess the histopathological changes. Paraffinized tissue sections (4–5 µm) were cut, examined, and photographed by an Olympus light microscope with an integrated camera.

### 2.6 Immunohistochemical study

Quantitative immune reactions using Nrf2 and cleaved caspase-3 antibodies were done according to the method described previously (Hassanen et al., 2023). Deparaffinized sections were mounted on positively charged glass slides and incubated with anti-cleaved caspase-3 antibody (Cat. #MC0123, Medaysis, United States, 1:2000) or anti-Nrf2 (GTX1013322, Genentex, Alton Pkwy, Irvine, CA, United States, 1:100).

The slides were investigated blindly using a light microscope at ×200 magnification. Five randomly selected images, one from each of five distinct fields per sample, were chosen to quantify the mean percentage of area displaying positive expression of cleaved caspase-3 and Nrf2. The Image-Pro Plus program version 6.0 (Media Cybernetics Inc., Bethesda, Maryland, United States) was used for histomorphometric evaluation.

### 2.7 Transmission electron microscopy (TEM)

Examination of ultra-histological features using TEM was performed as described previously (White and John, 2014; Woods and Stirling, 2019). Sites with cardiomyocytes longitudinally arranged were chosen. Sections from these areas were prepared using an ultramicrotome, placed on copper grids. The sections were subsequently examined using TEM.

### 2.8 IMI target prediction and pathway enrichment analyses

Screening of potential targets of IMI using *in silico* bioinformatics tools (GeneCards), Comparative Toxicogenomics Database (CTD, http://ctdbase.org/) (Davis et al., 2025), and DisGeNet, (http://www.disgenet.org) (Piñero et al., 2020). The genes involved in the pathogenesis of cardiotoxicity were screened using DisGeNet. We limited the species to “*Homo sapiens*.” A Venn diagram was created to determine the common targets involved in IMI-induced cardiotoxicity (https://bioinformatics.psb.ugent.be/webtools/Venn/), as described in (Aihaiti, 2021).

The intersected target genes from IMI and cardiotoxicity were submitted to Enrichr, https://maayanlab.cloud/Enrichr/(Kuleshov et al., 2016; Chen et al., 2013; Evangelista et al., 2023; Xie et al., 2021) to evaluate Gene Ontology (GO) enrichment analyses, including biological processes (BP), cellular components (CC), and molecular functions (MF). The Kyoto Encyclopedia of Genes and Genomes (KEGG) pathway, MSigDB Hallmark gene sets, and Reactome pathway enrichment analyses were conducted, and the top 10 pathways were graphically represented, providing functional annotation and classification for the target proteins, with a cutoff of *p < 0.05.*

### 2.9 Biochemical analysis

#### 2.9.1 Cardiac troponin I (cTnI) and creatine Kinase-MB (CK-MB)

Serum samples were used to determine cTnI and CK-MB concentrations using kits from Cusabio, China, and Spinreact, Spain, respectively, according to the manufacturers’ procedures.

#### 2.9.2 Assessment of oxidative stress biomarkers and proinflammatory cytokines

Commercial kits supplied by Biodiagnostics in Cairo, Egypt were utilized to evaluate cardiac catalase (Cat. # CA 25 17), reduced glutathione (GSH) (Cat. # GR 25 11), Thio barbituric acid reactive substances (TBARS) (Cat. # SD 25 29), and superoxide dismutase (SOD) (Cat. # SD 25 21), in accordance with the manufacturer’s protocol.

#### 2.9.3 Assessment of TNF-α and IL-6 in the heart homogenate

Cardiac interleukin-6 (IL-6) was assayed utilizing the Rat IL-6 ELISA Kit (SIGMA, United States), and tumor necrosis factor (TNF-α) was quantified utilizing the Rat TNF-α ELISA Kit (ORiGENE, United States) according to the manufacturer’s procedure.

#### 2.9.4 Western blotting and protein expression analysis

Protein expressions were investigated using Western blotting, as detailed previously. Briefly, proteins were extracted and quantified as described in (Badr et al., 2023). Proteins were separated by SDS-PAGE and then transferred to polyvinylidene difluoride (PVDF) membranes, which were supplied by Bio-Rad, a company based in Hercules, CA, United States. The membranes were first treated with primary antibodies at the required dilutions, followed by washing. TLR4, NF-κBp65, TGF β1, NLRP3, IL-1β, GSDMD, and GAPDH antibodies were applied at a 1:1000 dilution. In addition, JAK2 (Cat. # 3230), pJAK2 (Cat. # 3774), p-STAT3 (Cat. # 9131), and STAT3 (Cat. # 30835) were obtained from Cell Signaling Tech, Beverly, MA, United States at a 2:1000 dilution, and p-NF-κB p65 (sc-33020) was purchased from Santa Cruz (United States) and applied at 3:1000 dilution. GAPDH was used as an internal control. Data were quantified using densitometric analysis with the ImageJ software and then normalized to GAPDH levels, with the software being developed by the NIH, located at Bethesda, MD, United States.

### 2.10 Statistical analysis

The data were analyzed using GraphPad Prism V5, with a one-way analysis of variance (ANOVA) test, followed by a Tukey-Kramer *post hoc* analysis. Values of *P* less than 0.05 were deemed statistically significant.

## 3 Results

### 3.1 The effect of IMI with and without BBR-Lip on the myocardium histology in rats

**FIGURE 1:**
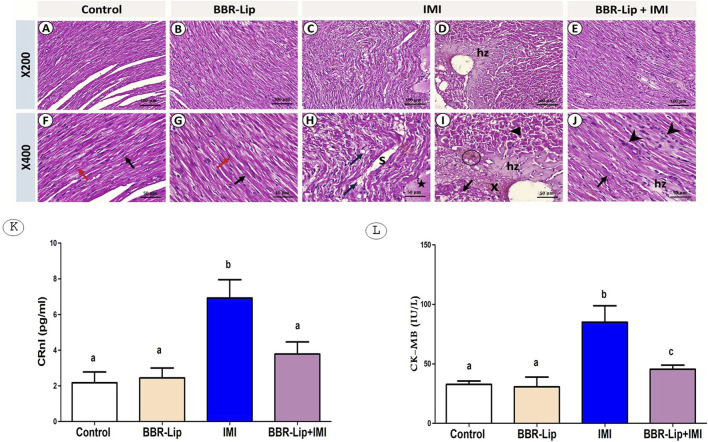
The Effect of Berberine Nanoliposomes (BBR-Lip) on Imidacloprid (IMI)-induced cardiotoxicity: Representative set of photomicrographs of sections stained with H and E from left ventricular walls of different experimental groups at two different magnifications: (A, B, C, (D) mag. ×200) with higher magnification at (F, G, H, (I) ×400), respectively. **(A,F)** Control group: **(A)** normal myocardium arrangement. **(F)** The striated cardiac fibers with branched patterns show multiple central oval euchromatic nuclei (black arrow). Delicate supporting tissue fills the minimal intercellular spaces with flat, darker nuclei of fibroblasts (red arrow). No interstitial edema or inflammatory cells were noticed. **(B,G)** BBR-Lip group: showing the same results as the control group, with no pathological alteration. **(C,H,D,I)** IMI group: **(C)** the fibers showing marked disorganization with lost regularity. **(H)** Multiple small sarcoplasmic vacuoles (blue arrows), large intercellular spaces (s), exudation, and tissue edema were apparent (star). **(D)** Disrupted cardiac muscle architecture. A large area of hyalinized fibers (hz) surrounded the large dilated ventricular vasculature. **(I)** The hyalinized fibers show a glassy appearance (hz). The myocytes either show hyper-eosinophilia with fragmentation (triangle), or degeneration with lost nuclei (black arrow). Marked vascular leakage was apparent (x). Notice the lipofuscin pigment (circle). (E, J BBR-Lip + IMI group: **(E)** showing restored architecture of fibers that appeared close to normal. **(J)** Most nuclei are oval, euchromatic, and central (black arrow). A few pathological changes are still observed as small focal hyalinized fibers (hz), and some inflammatory cell infiltrates (arrowheads). Effect of BBR-Lip administration on serum levels of **(K)** cardiac troponin (CTnI) and **(L)** creatine kinase-MB (CK-MB) in IMI-exposed rats. a, **(B)** Significantly different from the control and IMI groups, respectively, at *p* < 0.05. Similar symbol indicates non significance, different symbols indicate significance.

The control and BBR-Lip groups exhibited normal ventricular myocardium, with striated cardiac fibers arranged in a branched pattern, showing multiple central oval euchromatic nuclei. Cells are supported by delicate connective tissue endomysium, filling the minimal intercellular spaces with flat nuclei of fibroblasts. No interstitial edema or inflammatory cells were noticed (Figures 1A,B,F,G). Following IMI exposure, cardiac muscle architecture was disrupted, characterized by marked irregular fiber disorganization, multiple sarcoplasmic vacuoles, intercellular spaces, and tissue edema. Large areas of hyalinization with glassy shape, hyper-eosinophilia with fragmentation of myocytes, or cellular degeneration were apparent, and vascular leakage was obvious (Figures 1C,D,H,I). Co-treatment with BBR-Lip restored myocardial architecture to near-normal appearance. Most of the nuclei seemed to be oval, euchromatic, and central. Some focal hyalinized fibers and inflammatory cell infiltrates were present (Figures 1E,J). These results suggest the potential protective effects of BBR-Lip on cardiac tissues.

### 3.2 The effect of IMI and/or BBR-Lip on cardiotoxicity biomarkers

IMI significantly increased the levels of cardiac injury markers, cTnI and CK-MB, by about 3 and 2-fold compared to the control group (Figures 1K,L). However, co-administration of BBR-Lip significantly reduced IMI-induced increases in cTnI and CK-MB levels, demonstrating effective cardioprotective activity. BBR-Lip reduced both cTnI and CK-MB by approximately 50% as compared to the IMI group. It is worth mentioning that BBR-Lip treatment alone maintained cTnI and CK-MB levels comparable to control rats.

### 3.3 The effect of IMI and BBR-Lip administration on the myocardium examined by TEM

**FIGURE 2:**
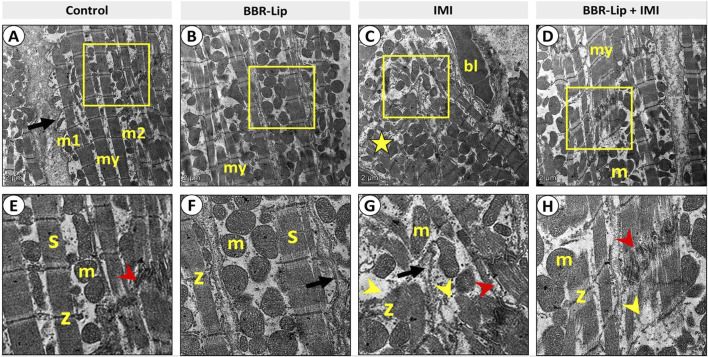
Representative set of electron micrographs of sections from left ventricular walls of different experimental groups at two different magnifications; **(A–D)** scale bar, 2 μm) with higher magnification at **(E–H)** respectively. **(A,E)** Control group. **(A)** The cardiac myocytes (my) are formed of sarcomeres with an identical banding pattern enclosed within a scalloped sarcolemma (arrow). The abundant mitochondria are either located sub-sarcolemma (m1) or located as rows separating the columns of sarcomeres (m2). **(E)**: The sarcomeres meet end-to-end at z-lines (z), while cardiac myocytes meet end-to-end at the junctional area, the intercalated disc (red arrowhead). **(B,F)** BBR-Lip group: showing the same histological arrangement as the control group without any pathological alteration. **(C,G)** IMI group. **(C)** Obvious disrupted architecture; most fibers are small and irregular with variable-sized mitochondria in between (star). The intercellular spaces show congested capillaries (bl). **(G)** The sarcolemma shows irregularity (arrow), intercalated discs are disrupted (red arrowhead), and most sarcomeres appear distorted (yellow arrowheads), with z-lines hardly observed (z). **(D,H)** BBR-Lip + IMI group: improved histological architecture of myocytes (my). Intercalated discs (red arrowheads), z-lines (z), and mitochondria (m) appeared close to normal. Some myocytes still show focal distortion of sarcomeres (yellow arrowheads). BBR-Lip: Berberine Nanoliposomes. IMI: Imidacloprid.

In this study, the control group showed that cardiomyocytes were formed of sarcomeres with identical banding patterns enclosed within a scalloped sarcolemma. Abundant mitochondria are either located sub-sarcolemmally or arranged in rows separating the columns of sarcomeres. Intact Z-lines and intercalated discs were obvious (Figures 2A,E). The BBR-Lip-treated group exhibited the same ultrastructural arrangement as the control group, without any pathological alterations (Figures 2B,F). After intoxication with IMI, there was apparent heterogeneity in myofibril patterns, with disintegration of myofilaments; most fibers were small and irregular, with irregular sarcolemma and variable-sized mitochondria. The intercellular spaces showed congested capillaries. Most Z-lines and intercalated discs were disrupted (Figures 2C, G). On the contrary, co-administration of BBR-Lip + IMI improved the histological architecture of myocytes. Intercalated discs, z-lines, and mitochondria appeared close to normal. Some myocytes showed focal distortion of sarcomeres (Figures 2D,H).

### 3.4 Target prediction and pathway enrichment analyses

**FIGURE 3:**
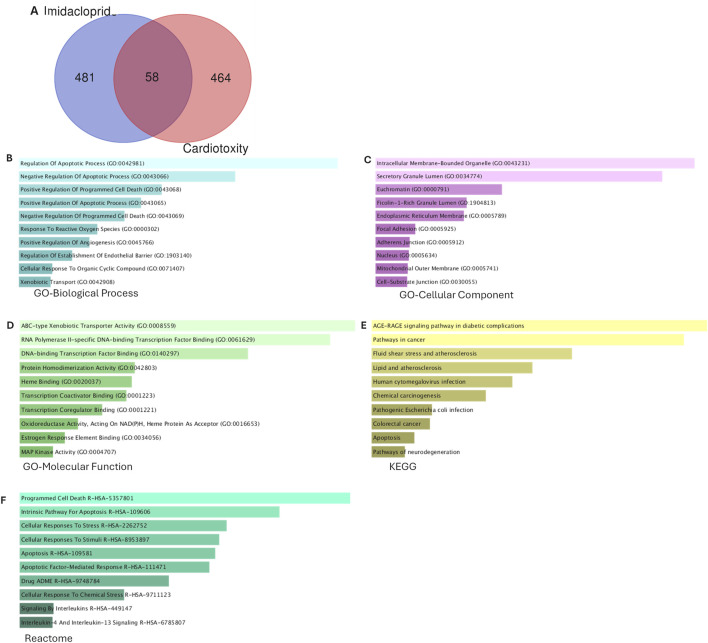
Ontology and pathway enrichment analysis of potential imidacloprid and cardiotoxicity target genes. Venn diagram of intersected genes **(A)**, bar graphs of ontological analysis of biological process **(B)**, cellular component **(C)**, molecular function **(D)**, KEGG pathway **(E)**, and reactome pathway **(F)**.

In this study, we identified 58 genes as potential targets of IMI-associated cardiotoxicity (Figure 3A; Supplementary Table S2). Target genes are mainly enriched in regulating apoptosis, programmed cell death, and response to oxidative stress in BP analysis (Figure 3B). The intracellular membrane-bounded organelles, granule lumen, and euchromatin are among the highly enriched in CC analysis (Figure 3C). The MF analysis revealed that xenobiotic transport activity, DNA transcription factor binding, protein homodimerization, oxidoreductase, estrogen response element binding, and MAPK activity were highly enriched (Figure 3D). To further investigate the potential underlying mechanism of IMI-mediated cardiotoxicity, a pathway model was identified through KEGG (Figure 3E) and Reactome (Figure 3F) pathway analyses. IMI is potentially implicated in cell death, cellular stress response, Receptor for Advanced Glycation End-Product (RAGE) signaling, cancer, atherosclerosis, and infection.

### 3.5 The impact of IMI with or without BBR-Lip on the oxidative stress markers in rat myocardium

**FIGURE 4:**
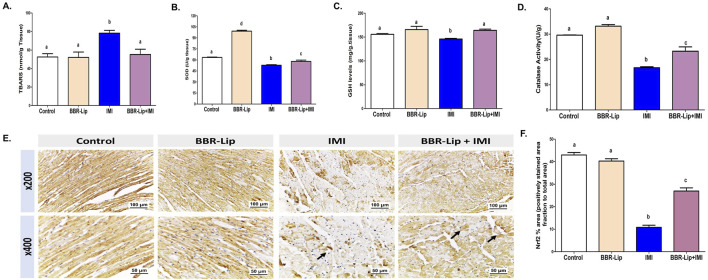
Effect of BBR-Lip administration on the levels of oxidative stress markers in IMI-exposed rats. The levels of **(A)** TBARS, **(B)** SOD, **(C)** GSH, and **(D)** catalase were quantified for the different groups. **(E)** A representative set of Nrf2-stained sections from the left ventricular walls of different experimental groups was quantified through IHC, at two different magnifications: (mag. X200 and X400). **(F)** Mean area percentages of IHC staining for Nrf2 for the different experimental groups. Values are mean ± SEM (n = 4–6). a, b: Significantly different from the control and IMI groups, respectively, at *p* < 0.05. *p* < 0.05 was evaluated using ANOVA followed by Tukey–Kramer as a *post hoc* test. Similar symbol indicates non significance, different symbols indicate significance. BBR-Lip: Berberine Nanoliposomes, GSH: reduced glutathione, IHC: immunohistochemistry, IMI: Imidacloprid, TBARS: Thiobarbituric Acid Reactive Substances, SOD: Superoxide dismutase.

Furthermore, the antioxidant defense status in cardiac tissue was evaluated by determining the levels of TBARS, SOD, GSH, and catalase activity. IMI treatment markedly increased TBARS levels and significantly reduced the levels of SOD, GSH, and catalase activity compared to the control group, indicating oxidative stress-induced damage (Figures 4A–D). IMI increased TBARS by about 1.5-fold and reduced the levels of both SOD and catalase by approximately 50% and that of GSH by 14%, as compared to the control group. Conversely, BBR-Lip co-treatment significantly decreased TBARS levels (by about 32%) and restored antioxidant parameters, as evidenced by the significant elevation in SOD and GSH (by about 1.15- fold) and catalase activity (by about 1.5-fold), as compared to IMI alone (Figures 4A–D). Rats treated with BBR-Lip alone showed significant upregulation of SOD and catalase levels, as compared to the control group, while the levels of TBARS and GSH were comparable to those of the control group.

Moreover, it is well known that Nrf2 plays a crucial role in cellular protection and survival by regulating the expression of antioxidant enzymes. Nrf2 immunoreactivity in the control and BBR-Lip groups showed high expression in most cardiomyocytes, indicated by a brown color (Figure 4E). The IMI group exhibited significantly weaker Nrf2 expression in most cardiomyocytes, except in a few areas (Figure 4E). IMI induced about 75% reduction in Nrf2 expression, as compared to the control group (Figure 4F). Co-administration of BBR-Lip + IMI displayed a more robust increase in Nrf2 expression compared to the IMI group, approximately 2.5-fold increase. However, this elevation did not reach the values of the control group, remaining significantly lower (Figures 4E,F).

### 3.6 The impact of IMI and BBR-Lip on the expression of inflammatory proteins and TLR4/NLRP3 inflammasome pathway in rat myocardium

**FIGURE 5:**
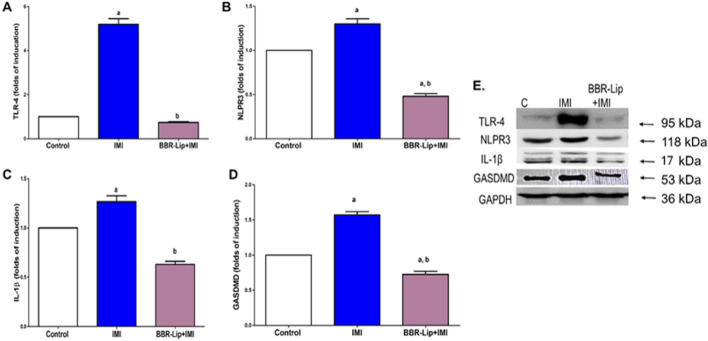
Effect of BBR-Lip administration on the TLR4/NLRP3 Inflammasome pathway and inflammatory markers in the myocardium of IMI-exposed rats. The effect of BBR-Lip administration on the TLR4/NLRP3 inflammasome pathway was investigated through the determination of the levels of **(A)** TLR4, **(B)** NLRP3, **(C)** IL-1β, and **(D)** GSDMD. **(E)** A representative Western blotting diagram for quantified proteins. Loading control image of GAPDH is reused for all samples run on the same gel/membrane for illustrative purposes. Values are mean ± SEM (n = 3). a, b: Significantly different from the control and IMI groups, respectively, at *p* < 0.05. *p* < 0.05 was evaluated using ANOVA followed by Tukey–Kramer as a *post hoc* test. Similar symbol indicates non significance, different symbols indicate significance. TLR4: Toll-like receptor 4, BBR-Lip: Berberine Nanoliposomes. IMI: Imidacloprid, GSDMD: Gasdermin.

Exposure to IMI resulted in a marked upregulation of TLR4, NLRP3, IL-1β, and GSDMD expressions, by an average of 5-, 1.3-, 1.25-, and 1.5-fold, respectively, compared to the control group. This suggests a marked contribution of the inflammasome signaling cascade in IMI-induced cardiotoxicity (Figures 5A–E). The Western blotting chart shows the elevated protein expression levels of these markers in IMI-exposed rats (Figure 5E). Interestingly, co-administration of BBR-Lip significantly blunted IMI-induced inflammasome activation as evidenced by attenuation of the expression of TLR4, NLRP3, IL-1β, and GSDMD, compared to the IMI group.

**FIGURE 6:**
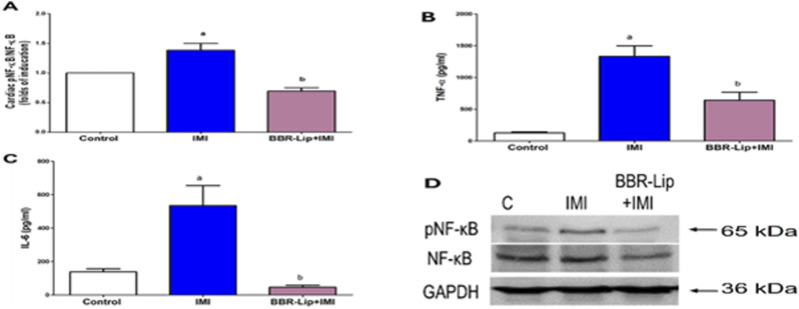
Effect of BBR-Lip administration on the inflammatory markers expression in the myocardium of IMI-exposed rats. The effect of BBR-Lip administration on the inflammatory markers was investigated through the determination of the cardiac levels of **(A)** pNF-κB/NF-κB and the serum levels of TNF-α **(B)** and IL-6 **(C)**. **(D)** A representative Western blotting diagram for NF-κB and pNF-κB quantified in the myocardium. Control image of GAPDH is reused for illustrative purposes. Values are mean ± SEM (n = 3). a, b: Significantly different from the control and IMI groups, respectively, at *p* < 0.05. *p* < 0.05 was evaluated using ANOVA followed by Tukey–Kramer as a *post hoc* test. Similar symbol indicates non significance; different symbols indicate significance. BBR-Lip: Berberine Nanoliposomes. IMI: Imidacloprid.

Additionally, IMI induced a marked increase in NF-κB phosphorylation (1.5-fold) (Figure 6A), which was accompanied by a significant increase in cardiac TNF-α (14-fold) and IL-6 levels (2.8-fold), compared to the control group (Figures 6B,C). Co-administering BBR-Lip with IMI effectively reduced the inflammatory responses, as evidenced by a significant decrease in the phosphorylation of NF-κB (by 50%), as well as IL-6 (by 80%) and TNF-α levels (by 58%), compared to the group treated with IMI alone (Figure 6). The Western blot chart shows a pronounced increase in pNF-κB expression in the IMI group, followed by a decrease upon BBR-Lip administration (Figure 6D).

### 3.7 IMI induces JAK/STAT phosphorylation and TGF-β protein expression

**FIGURE 7:**
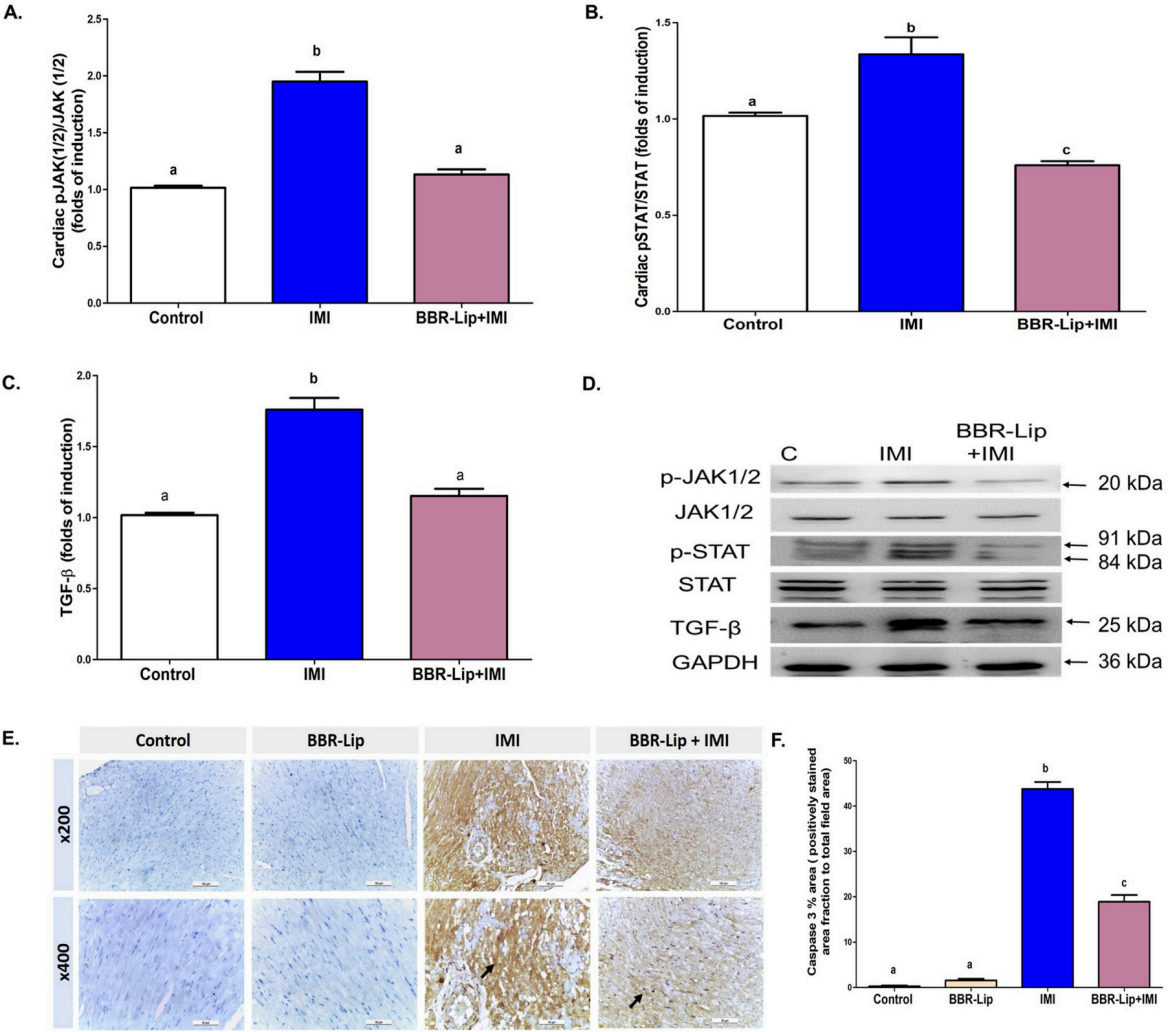
Effect of BBR-Lip administration on the phosphorylation of JAK/STAT and the expression of TGF-β and caspase-3 in the myocardium of IMI-exposed rats. The cardiac levels of **(A)** pJAK(1/2)/JAK(1/2), **(B)** pSTAT/STAT, and **(C)** TGF-β were quantified for the different groups. **(D)** A representative Western blotting diagram for JAK (1/2), pJAK (1/2), STAT, pSTAT, and TGF-β. Values are mean ± SEM (n = 3). Loading control image of GAPDH for all samples run on the same gel/membrane is reused for illustrative purposes. **(E)** Representative set of caspase 3-stained sections from the left ventricular walls of different experimental groups quantified through immunohistochemistry (IHC). **(F)** Mean area percentages of IHC staining for caspase 3 for the different experimental groups. Values are mean ± SEM (n = 6). a, b: Significantly different from the control and IMI groups, respectively, at *p* < 0.05. *p* < 0.05 was evaluated using ANOVA followed by Tukey–Kramer as a *post hoc* test. Similar symbol indicates non significance; different symbols indicate significance. BBR-Lip: Berberine Nanoliposomes, IMI: Imidacloprid, TGF-β: Transforming growth factor-β.

IMI administration significantly elevated the phosphorylation of JAK1/2 and STAT (by an average of 2- and 1.3-fold, respectively), as well as the expression of TGF-β (by 1.75-fold), when compared to control rats, indicating the activation of this pro-fibrotic and inflammatory signaling axis (Figures 7A–C). The Western blotting chart shows a pronounced increase in p-JAK1/2, p-STAT, and TGF-β expression in the IMI group and the subsequent decrease upon BBR-Lip administration (Figure 7D). In contrast, co-treatment with BBR-Lip significantly mitigated the IMI-induced increases in p-JAK1/2, p-STAT, and TGF-β levels by 37%, 40%, and 32%, respectively, which indicates the efficacy of BBR-Lip to suppress JAK/STAT phosphorylation and downstream fibrotic signaling in the myocardium. These data demonstrate that BBR-Lip alleviates IMI-induced cardiac injury, at least in part, by downregulating the activation of the JAK/STAT pathway and the expression of TGF-β, and hence, attenuating pro-inflammatory and pro-fibrotic states.

### 3.8 The impact of IMI with or without BBR-Lip on the immunohistochemical expression of cleaved Caspase-3 in the rat myocardium

It has been established that cardiomyocyte apoptosis is a critical mechanism in the early cardiac response to toxins. Results of cleaved caspase-3 immunoreactivity are represented in Figure 7E. The control and BBR-Lip groups displayed no significantly visible precipitation of caspase 3 in cardiomyocytes, indicating no apoptotic activity (Figure 7E). Exposure to IMI induced a substantial increase in caspase-3 activity, as evidenced by prominent brown sarcoplasmic and nuclear expression in the majority of cardiomyocytes (Figure 7E), indicating a switch to an apoptotic state. Co-administration of BBR-Lip + IMI dramatically (*P* < 0.05) reversed caspase-3 expression compared to the IMI group (Figures 7E,F).

## 4 Discussion

IMI is the most widely used neonicotinoid globally, accounting for almost 42 percent of the total market (Jeschke et al., 2005). Despite the known claim that these insecticides are not very harmful to mammals, extended exposure to IMI can have serious negative health effects on both humans and animals (Katić et al., 2021; Perananthan et al., 2021). This exposure most likely occurs when tainted dust and pollen are inhaled or contaminated food and water are consumed (Wu et al., 2001; Zhang and Lu, 2022). Despite its relatively low mammalian toxicity, cases of acute poisoning and fatalities have been reported. The acute oral LD50 values are 450 mg/kg in rats and 150 mg/kg in mice (Tomlin, 1994; Lee Chao and Casida, 1997). The NOAEL, or no observed adverse effect level, has been determined to be 14 mg/kg/day in rats, as reported previously (Omar et al., 2023). No studies were found that humans chronically exposed to IMI, and the chronic dietary reference dose (RfD) in humans, based on studies in rats, has been calculated at 0.057 mg/kg/day, with a NOAEL of 5.7 mg/kg/day (Zhang et al., 2019).

Although hepatotoxicity, nephrotoxicity, neurotoxicity, mutagenicity, and immunotoxicity have been previously reported to occur in laboratory animals following IMI exposure (Katić et al., 2021; Hassanen et al., 2023), reports on the effects of IMI exposure on cardiac tissues are very limited. Two case reports link tachycardia, bradycardia, arrhythmias, and cardiac arrest in humans to IMI acute exposure (Shadnia and Moghaddam, 2008; Perananthan et al., 2021). However, no single controlled study in mammals has fully assessed IMI-induced cardiotoxicity, defined histopathological and biochemical changes, and explored the possible mechanisms implicated. IMI target prediction and pathway analysis suggested that cell death and inflammation are among the top-enriched pathways.

In line with our hypothesis that IMI induces cardiotoxicity through oxidative stress and inflammation, IMI was administered orally for 30 days to elicit cardiotoxicity in rats. In comparison with the control group, IMI-induced cardiotoxicity was supported with a marked increase in CTnI and CK-MB, two well-established biomarkers of myocardial damage, as well as significant histopathological changes in cardiac tissue, demonstrated by both light and TEM. Compared to the IMI group, the administration of 10 mg/kg BBR-Lip resulted in a significant improvement in both cardiotoxicity parameters and histopathological alterations, which showed centrally located oval euchromatic nuclei, with only a slight presence of focal hyalinized fibers and inflammatory cell infiltrates. This suggests that BBR-Lip possesses cardioprotective properties against IMI-induced intoxication. These results are in line with earlier studies reporting the cardioprotective effects of BBR (50 mg/kg) against doxorubicin-induced cardiomyopathy in rats by improving myocardial architecture (Wang et al., 2023; Li et al., 2022).

Furthermore, oxidative stress is involved in multi-organ toxicity induced by IMI (Lesser, 2006). In our study, IMI induced a significant oxidative stress response, reflected by elevated TBARS and marked reductions in antioxidant defenses including GSH, SOD, and catalase. This agrees with earlier studies showing oxidative stress as a primary cause of IMI-induced toxicities in rats received 45 mg/kg/day orally for 30 days (El Gazzar et al., 2025; Mudgal et al., 2023; Hassanen et al., 2022). Consequently, the histopathological alterations observed in the IMI group can be attributed to oxidative stress, as evidenced by depletion of antioxidant defenses.

The transcription factor Nrf2 is also essential for preserving redox homeostasis. Oxidative stress-induced Keap1 activation inhibits Nrf2 nuclear translocation, where it interacts with antioxidant response elements (AREs), inducing the expression of antioxidant genes encoding SOD, GSH, and HO-1. Thus, supporting the cardiac defense against oxidative stress, potentially via reducing lipid peroxidation, mitochondrial dysfunction, and cardiomyocyte apoptosis (Tripathi et al., 2024; Kang, 2020). Accordingly, our findings showed a significant downregulation of cardiac Nrf2 expression following IMI administration, and there was also a noticeable drop in antioxidants GSH, SOD, and catalase, shifting the equilibrium toward prooxidants, inducing oxidative stress and cardiotoxicity. Consistent with our results, previous studies have linked IMI-induced Nrf2 suppression in hepatocytes to a weakened antioxidant defense system within the cells, ultimately resulting in mitochondrial dysfunction and hepatocyte apoptosis (Deng et al., 2022). On the other hand, Nrf2 activation has been documented to reduce cardiac injury by reducing inflammatory cytokines and restoring antioxidant enzyme levels (Wang et al., 2023; Bai et al., 2015).

Furthermore, exposure to IMI has been associated with increased inflammation in multiple organ systems (Xie et al., 2024; Shao et al., 2020). Our results demonstrated that IMI elicits a pronounced inflammatory reaction in cardiac tissue evidenced by marked elevations in the protein expression of pro-inflammatory markers, including NF-κB and pNFκB p65, the principal transcription factor that governs inflammation. NF-κB is known to induce the transcription of various inflammatory mediators, including IL-6 and TNF-α, as well as TGF-β1, which showed a significant elevation in the IMI group (Mudgal et al., 2023; Miao et al., 2022). The link between NF-κB activation and increased production of ROS, exacerbating cellular damage, has been established previously (Hassanen et al., 2023; Miao et al., 2021).

Furthermore, IMI activated the TLR4/NLRP3 inflammasome pathway. In the IMI-exposed group, cardiac tissue TLR4 expression was significantly higher compared to the control group, supporting earlier data that showed TLR4/NF-κB signaling crosstalks in cardiac tissues and further promotes the production of pro-inflammatory cytokines (Lan et al., 2020; Kim et al., 2023). TLR4 is a pattern recognition receptor that, once activated, induces a massive inflammatory response and activates NLRP3 inflammasome pathway (Badr et al., 2023). This results in caspase-1 recruitment and cleavage of pro-IL-1β and pro-IL-18 into their active, pro-inflammatory forms. The active IL-1β is subsequently secreted from the cell to interact with its cognate receptor in an autocrine or paracrine manner, ultimately resulting in increased expression and activation of NF-κB and other inflammatory cytokines. NLRP3 inflammasomes play a considerable role in cardiomyocyte death, fibrosis, and hypertrophy (Toldo et al., 2022; Mo et al., 2025; Ding et al., 2022). Reviewing literature, no data has been found investigating the role of inflammasome activation in IMI-induced cardiotoxicity. Our study provides new mechanistic insight into how IMI induces cardiac injury, showing that activation of the TLR4/NLRP3 inflammasome pathway might have a significant role. This was supported by the marked upregulation of TLR4, NLRP3, and IL-1β protein expression in the hearts of IMI-exposed rats. Our findings are consistent with the data obtained by Liu et al. (2023) which showed that IMI triggers the NLRP3/IL-1β/IL-18 and pyroptosis pathways in epithelioma papulosum cyprini cells, when cells were treated with IMI across a wide range of concentrations (15–1000 mg/L) (Liu et al., 2023).

Conversely, our results demonstrate that the BBR-Lip oral dose significantly reduced the IMI-induced oxidative damage in cardiac tissue, as shown by normalized Nrf2 expression, GSH, SOD, catalase, and TBARS levels. A previous study reported that BBR increased SOD by enhancing Nrf2 signaling, which in turn prevented oxidative stress (Feng et al., 2019). Besides the indirect activation of antioxidant enzymes, BBR possesses an antioxidant effect, mainly via direct free radical scavenging (García-Muñoz et al., 2024; Li et al., 2014). This finding aligns with earlier research that investigated the cardioprotective effects of BBR (An, 2022; Wang et al., 2023). The current study supported the idea that administering BBR-Lip activated the Nrf2 signaling pathway and restored antioxidant enzyme activities, possibly adding to its cardioprotective effects. Additionally, all pro-inflammatory cytokines and TLR4/NLRP3 inflammasomes had their expression downregulated by BBR-Lip. We propose that its anti-inflammatory activity can be attributed to its ability to suppress the ROS-mediated activation of NF-κB. Furthermore, recent research has shown that Nrf2 and inflammasome signaling are reciprocal. By blocking ROS and NF-κB signaling, Nrf2 can inhibit inflammasome activation. Conversely, chronic inflammation reduces Nrf2 expression via an IL-1β-mediated mechanism, thereby weakening the antioxidant response and exacerbating oxidative damage (Hennig et al., 2018).

IMI not only activated the inflammatory pathways but also upregulated the expression of caspase-3, an apoptotic executor, and GSDMD, a pyroptosis marker as well. It is known that NLRP3 inflammasome activation can induce pyroptosis through GSDMD activation. IMI-induced NLRP3/GSDMD activation and pyroptosis have been previously reported in macrophages (Zhang et al., 2023). On the other hand, apoptosis can be induced by various signals, including TNF-α binding to death receptors, activating the extrinsic apoptotic pathway, and ROS-induced mitochondrial injury, which activates the intrinsic apoptotic pathway (Zhang et al., 2019). IMI was previously found to induce apoptosis in rat testes (Saber et al., 2021) and neurons (Chung et al., 2023). Both pyroptosis and apoptosis were found to contribute simultaneously to cardiac injury in various cardiac insults, including ischemia-reperfusion (Ding et al., 2023) and LPS toxicity (Wen et al., 2023). Thus, our study gives evidence of the upregulation of both apoptosis and pyroptosis in the heart of IMI-exposed rats. Both markers were downregulated by BBR-Lip, whose inhibitory effects on both pyroptosis (Li et al., 2025) and apoptosis (Liao et al., 2018) was previously reported.

Lastly, a key element of the myocardial response to various cardiac insults is the JAK/STAT signaling pathway (Chen et al., 2021; Boengler et al., 2008; Pang et al., 2023; Barry et al., 2007). A variety of biological processes, including immunity, cellular proliferation, apoptosis, and differentiation, are regulated through the activation of the JAK/STAT signalling pathway, which is recognized as a complex signalling system (Js et al., 2004). Whether STAT3 has positive, negative, or a combination of effects depends on the degree of its activation in the cardiac tissue. The physiological and constitutive activation of STAT3 leads to enhanced cardiac survival by increasing the expression of anti-apoptotic genes like Bcl-2 and Bcl-xL. Conversely, excessive STAT3 expression in the heart has been associated with pathological hypertrophy (Chen et al., 2021; Harhous et al., 2019). The JAK/STAT signaling pathway is known to promote inflammation (Wen et al., 2023; Li et al., 2025). Data regarding its possible role in increasing the expression of various inflammatory mediators, particularly IL-6 (Ridker and Rane, 2021) and TNF-α (Wang et al., 2021) are available. Moreover, a strong relationship exists between JAK/STAT activation and TGF-β which has been identified in previous research as a strong activator of the JAK/STAT pathway (Montero et al., 2021), and the activated STAT has binding sites in the promoter region of the TGF-β gene, through which STAT can directly further activate TGF-β gene transcription (Ogata et al., 2006). In addition, evidence exists of JAK/STAT interaction with NLRP3; Zhu et al. reported that the inhibition of NLRP3 accompanies the inhibition of STAT (Zhu et al., 2021). This suggests a direct effect of JAK/STAT on NLRP3 activation. This study documents IMI-induced JAK/STAT phosphorylation in cardiac tissues; there is no previous research associating neonicotinoids with JAK/STAT activation in mammalian hearts. BBR-Lip was found to inhibit IMI-induced phosphorylation of JAK/STAT. BBR-induced inhibition of JAK/STAT3 phosphorylation was previously documented in macrophages and other cells (Kim et al., 2021; Long, 2025; Haftcheshmeh et al., 2022; Li et al., 2020), but not in the heart. Although the current study comprehensively characterized the toxic mechanisms of IMI-induced cardiotoxicity through biochemical and molecular analyses, it is important to acknowledge that direct quantification of IMI concentrations in serum and cardiac tissue was not performed. This represents a methodological limitation that should be addressed in future investigations.

## 5 Conclusion

This study elucidates the mechanisms underlying the cardiotoxic effects of IMI in rats and confirms our hypothesis that oxidative stress and inflammation play central roles. The molecular mechanisms involve increased oxidative stress markers, accompanied by a reduction in Nrf2 expression and increased inflammation via the activation of NLRP3-inflammasome pathway. Additionally, upregulation of JAK/STAT and TGF-β signaling contributes to the pro-fibrotic effects of IMI. The study also investigated how administration of BBR-Lip can protect against IMI-induced cardiotoxicity by restoring antioxidant capacity, suppressing pro-inflammatory mediators. These results suggest that BBR-Lip represents a promising remedy for those at high risk of IMI-induced cardiotoxicity.

## Data Availability

The original contributions presented in the study are included in the article/Supplementary Material, further inquiries can be directed to the corresponding author.
