# Supplementary figures and images for: Imidacloprid exposure in rats induces cardiac inflammatory response through activating TLR4/NF-κB/NLRP3 and JAK/STAT signaling pathways: focus on the berberine-loaded nanoliposomes
Source: Front Toxicol. 2026 Jan 5;7:1701021. doi: 10.3389/ftox.2025.1701021 (PMC12812405; doi:10.3389/ftox.2025.1701021)

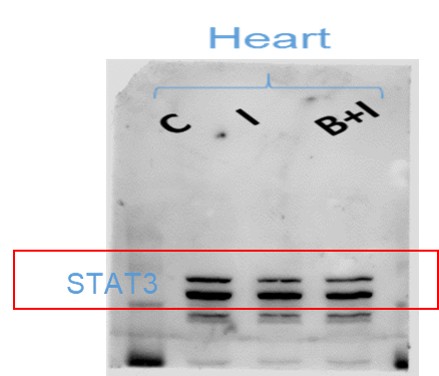

Supplement: Supplementary file 1 [file Image3.jpeg]

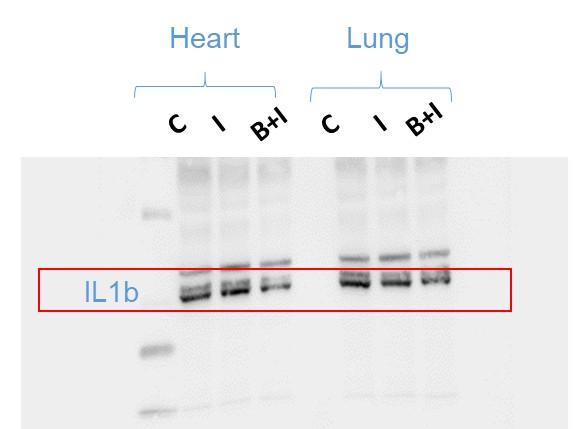

Supplement: Supplementary file 3 [file Image9.jpeg]

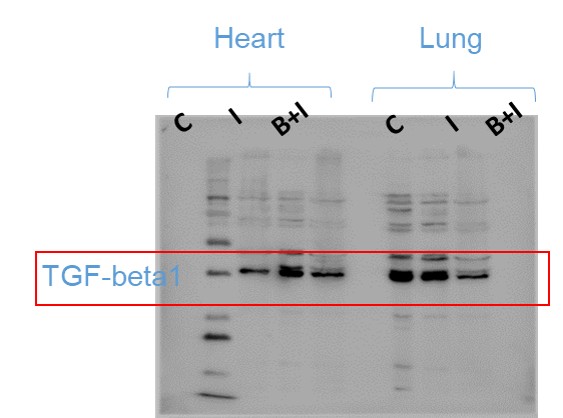

Supplement: Supplementary file 4 [file Image1.jpeg]

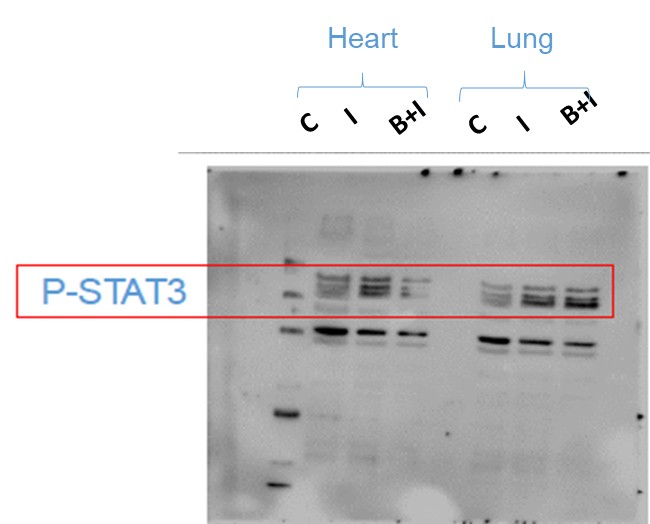

Supplement: Supplementary file 5 [file Image4.jpeg]

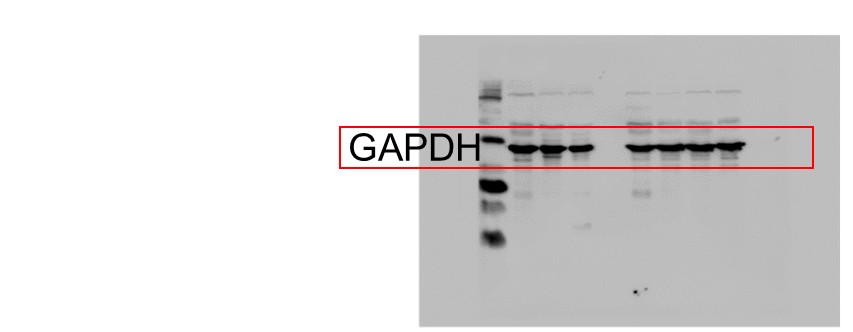

Supplement: Supplementary file 6 [file Image7.jpeg]

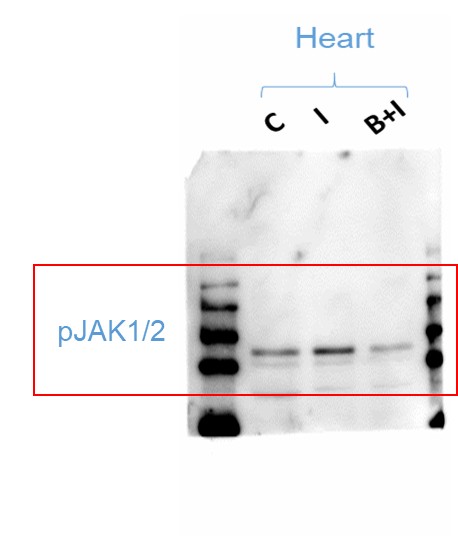

Supplement: Supplementary file 7 [file Image2.jpeg]

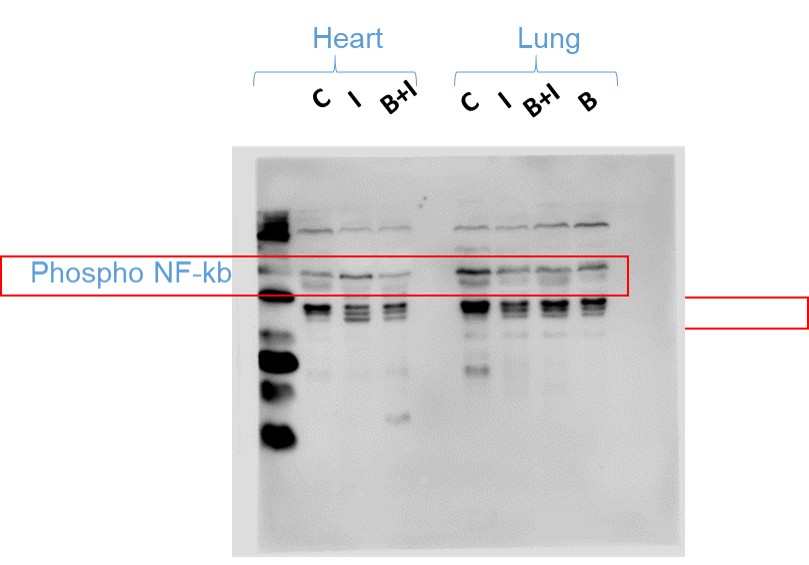

Supplement: Supplementary file 8 [file Image5.jpeg]

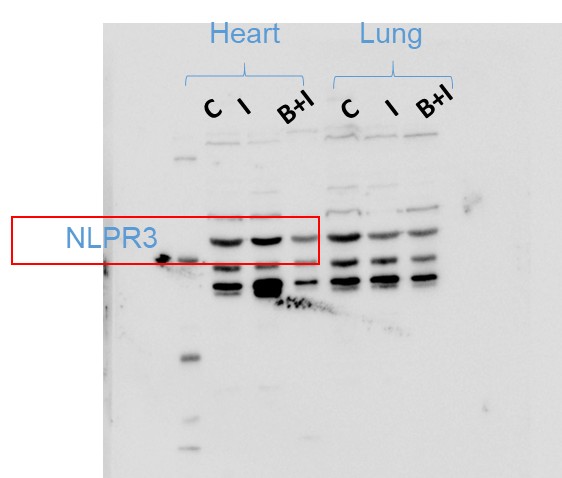

Supplement: Supplementary file 9 [file Image10.jpeg]

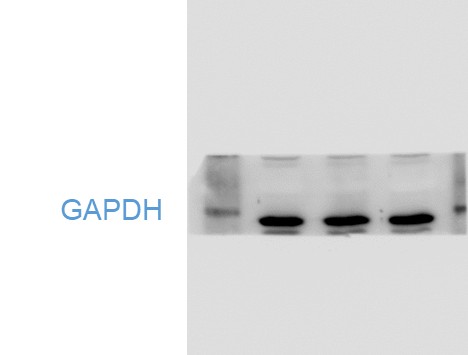

Supplement: Supplementary file 10 [file Image12.jpeg]

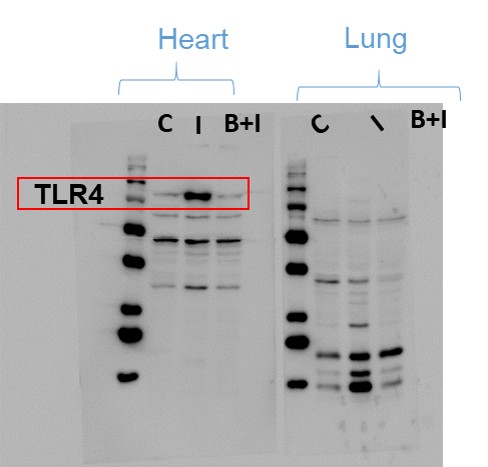

Supplement: Supplementary file 11 [file Image11.jpeg]

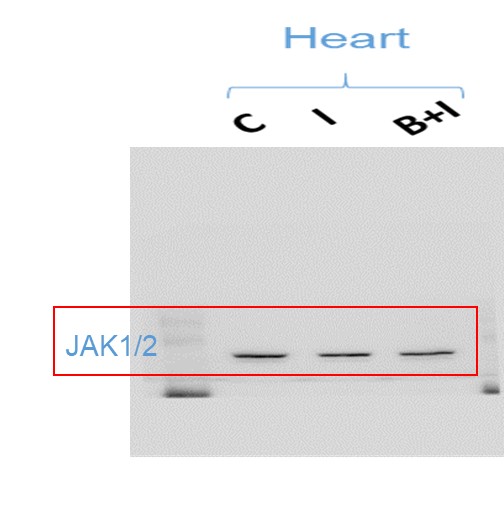

Supplement: Supplementary file 13 [file Image13.jpeg]

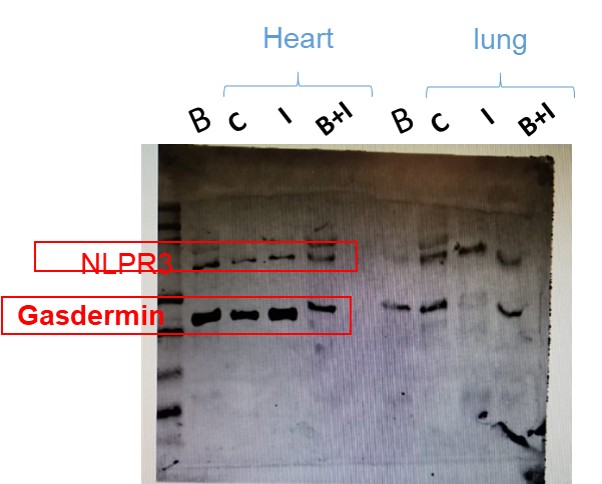

Supplement: Supplementary file 14 [file Image8.jpeg]

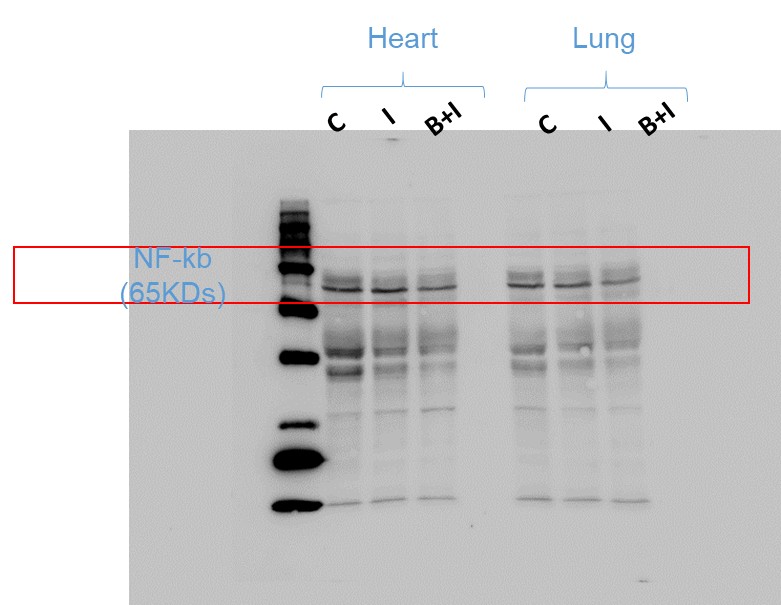

Supplement: Supplementary file 17 [file Image6.jpeg]
