# Supplementary material for: Imidacloprid exposure in rats induces cardiac inflammatory response through activating TLR4/NF-κB/NLRP3 and JAK/STAT signaling pathways: focus on the berberine-loaded nanoliposomes
Source: Front Toxicol. 2026 Jan 5;7:1701021. doi: 10.3389/ftox.2025.1701021 (PMC12812405; doi:10.3389/ftox.2025.1701021)
Supplement: Supplementary file 2 [file Table1.docx]

**Supplementary Materials**

**The Results of a Pilot Study Using Two Different Doses of Imidacloprid (IMI):**

Two doses of IMI; 22.5 and 45 mg/kg were used. Hearts and serum were collected. The serum was used for assessing cardiac injury markers; cardiac troponin (cTnI) and creatine kinase-MB. The cardiac homogenate was used for assessment of oxidative stress markers, thiobarbituric acid reactive substances (TBARS) and the antioxidant enzyme superoxide dismutase (SOD).

The low dose of IMI was not significantly different from the control with referral to the assessed parameters, while the higher dose showed a significant difference from both the control and the lower dose groups. That IMI dose of 45mg/kg was selected to elucidate and examine its cardiotoxicity, as well as the potential berberine cardioprotective one.

**Supplementary Table** **1**: **The Results of a Pilot Study Using Two Different Doses of Imidacloprid (IMI):**

| **Group** | **cTnI** | **CK-MB** | **SOD** | **TBARS** |
| --- | --- | --- | --- | --- |
| **Control** | 2.05 ± 0.5 | 32.25 ± 3.6 | 63.35 ± 0.6 | 56.35 ± 4.4 |
| **IMI (22.5 mg/kg)** | 1.5 ± 0.18 | 33.25± 3.2 | 62.93 ± 1.66 | 62.75 ± .2.2 |
| **IMI (45 mg/kg)** | 4.8 ± 1.04 **^a,^** **^b^** | 84.1 ± 8.8 **^a,^** **^b^** | 55.08 ± 0.7 **^a, b^** | 78.34 ± 3.2 **^a,^** **^b^** |

a, b: Significantly different from the control and IMI 22.5 mg/kg groups, respectively, at p < 0.05 using One-Way Anova and Tukey-Kramer multiple comparison test, n=4.
