# Supplementary material for: Imidacloprid exposure in rats induces cardiac inflammatory response through activating TLR4/NF-κB/NLRP3 and JAK/STAT signaling pathways: focus on the berberine-loaded nanoliposomes
Source: Front Toxicol. 2026 Jan 5;7:1701021. doi: 10.3389/ftox.2025.1701021 (PMC12812405; doi:10.3389/ftox.2025.1701021)
Supplement: Supplementary file 12 [file Table2.docx]

Supplementary Table 2 (S2):

**Cardiotoxity and Imidacloprid (58 genes)**

| **Name** |
| --- |
| CERNA3 |
| HMGCR |
| XDH |
| TKT |
| GSR |
| HSPB1 |
| ABCG2 |
| HMOX1 |
| PPARA |
| METTL134 |
| GJA1 |
| TIMP3 |
| MAPK1 |
| MB |
| NQO1 |
| TSPO |
| FAS |
| BCHE |
| BCL2 |
| THRB |
| MAPK8 |
| AGT |
| SLC2A4 |
| SMAD5-AS1 |
| ABCB1 |
| AHR |
| PGR |
| VCL |
| CYP19A1 |
| GABRB1 |
| TNF |
| CASP9 |
| CAT |
| MPO |
| CIRBP |
| SOD2-OT1 |
| CTNNB1 |
| TMX2-CTNND1 |
| ABCC1 |
| IL-1β |
| BAG3 |
| CYP2E1 |
| LINV01672 |
| ESR1 |
| TJP1 |
| VEGFA |
| SIRT1 |
| CDKN2A |
| BAX |
| GPT |
| EMSLR |
| CYP3A4 |
| GATA4 |
| HSP910AA1 |
| CASP3 |
| CHKB-CPT1B |
| SOD1 |
| POR |

A list of studied genes function was provided as separate csv file, named as List_1_DAVID Functional Annotation Table.

The accession number of the different genes was also provided as separate csv files, named as DAVIDConversion_List_1_GENBANK_ACCESSION.
